# Milk exosomes are bioavailable and distinct microRNA cargos have unique tissue distribution patterns

**DOI:** 10.1038/s41598-018-29780-1

**Published:** 2018-07-27

**Authors:** Sonia Manca, Bijaya Upadhyaya, Ezra Mutai, Amy T. Desaulniers, Rebecca A. Cederberg, Brett R. White, Janos Zempleni

**Affiliations:** 1 Department of Nutrition and Health Sciences, University of Nebraska-Lincoln, 316 Leverton Hall, Lincoln, NE 68583-0806 USA; 2 Department of Animal Science, University of Nebraska-Lincoln, A224j Animal Science Building, 3940 Fair Street, Lincoln, NE 68583-0908 USA

## Abstract

Exosomes participate in cell-to-cell communication, facilitated by the transfer of RNAs, proteins and lipids from donor to recipient cells. Exosomes and their RNA cargos do not exclusively originate from endogenous synthesis but may also be obtained from dietary sources such as the inter-species transfer of exosomes and RNAs in bovine milk to humans. Here, we assessed the bioavailability and distribution of exosomes and their microRNA cargos from bovine, porcine and murine milk within and across species boundaries. Milk exosomes labeled with fluorophores or fluorescent fusion proteins accumulated in liver, spleen and brain following suckling, oral gavage and intravenous administration in mice and pigs. When synthetic, fluorophore-labeled microRNAs were transfected into bovine milk exosomes and administered to mice, distinct species of microRNAs demonstrated unique distribution profiles and accumulated in intestinal mucosa, spleen, liver, heart or brain. Administration of bovine milk exosomes failed to rescue *Drosha* homozygous knockout mice, presumably due to low bioavailability or lack of essential microRNAs.

## Introduction

Virtually every living cell synthesizes and secretes exosomes into the extracellular space^1,2^. Exosomes play an essential role in cell-to-cell communication, which is achieved through the transfer of exosome cargos, such as various species of RNAs, lipids and proteins, from donor cells to adjacent or distant recipient cells^1,3^. In recipient cells, exosome cargos regulate gene expression and metabolism. The roles of microRNAs in communication through exosomes has been particularly well characterized because microRNAs regulate the expression of more than 60% of mRNAs in humans and loss of microRNA maturation in *Drosha* knockout mice results in premature lethality^4^. MicroRNAs have been implicated in many, if not all, physiological and pathological conditions in humans^5–7^. For example, adipocyte-derived exosomes participate in transforming growth factor-β dysregulation in hepatocytes and obesity-related liver disease; these effects are facilitated by exosome-dependent shuttling of microRNAs from donor to recipient cells^8,9^. Notwithstanding the great importance of microRNA cargos in exosome-mediated communication, protein cargos may also play important roles in human physiology and pathology. For example, exosomes appear to participate in the spread of β-amyloid and α-synuclein in the propagation and diagnosis of Alzheimer’s Disease and Parkinson’s Disease^10–12^.

Previously, we provided evidence that exosomes and their cargos not only originate from endogenous synthesis but may also be obtained from dietary sources, including the transfer of bovine milk exosomes across species boundaries^13–17^. Specifically, we demonstrated that human intestinal and vascular endothelial cells, as well as rat intestinal cells, transport bovine milk exosomes into the cytoplasm by endocytosis and release their microRNA cargos across the basolateral membrane in cell cultures. Independent laboratories have corroborated these findings, including studies in mouse models^18–21^. Moreover, pigs-derived exosomes were demonstrated to be able to increase intestinal cell proliferation and development of intestinal tract in mice^22^.

The cargo of dietary exosomes appears to be biologically active. For example, microRNAs in bovine milk exosomes decreased the expression of genes in circulating immune cells in human feeding studies and in human kidney cell cultures^13^ and miR-148a in human milk downregulated the expression of DNA methyltransferase 1 in normal human colon cells and human leukemia cells^21^. Dietary microRNAs, encapsulated in milk exosomes, are protected against degradation by low pH, RNases and treatment that mimics digestion in the gastrointestinal tract^23–25^. Dietary depletion of exosomes and their cargos result in a phenotype of aberrant metabolism of purines as well as impaired spatial learning and memory in preliminary studies of humans and mice^26,27^. These observations raise concerns regarding infant nutrition by using formulas, because the content of microRNAs is lower compared to human milk^23^. The regulation of genes and metabolism by bovine milk exosomes and their cargos across species boundaries suggests that these compounds might represent novel classes of bioactive food compounds, as defined by the National Cancer Institute as “a type of chemical found in small amounts in plants and certain foods […]. Bioactive compounds have actions in the body that may promote good health and they are being studied in the prevention of […] diseases”^28^. That said, milk might have effects detrimental to health, although adverse effects of milk have not been formally linked with exosomes^29,30^. Finally, bovine milk exosomes are considered scalable vehicles for the delivery of unstable or poorly bioavailable drugs^19,20^.

Despite the great interest in bovine milk exosomes and their enormous potential in nutrition and pharmacology, we know little about the bioavailability and distribution of bovine milk exosomes in non-bovine species. Notable exceptions include evidence that (i) macrophages rapidly eliminate foreign exosomes in mice, (ii) bovine exosomes can be found in murine tissues following oral administration, and (iii) human intestinal cells transport distinct species of microRNAs, encapsulated in bovine milk exosomes, with varying efficacy^15,19,31^. Here, we provide evidence that the bioavailability and distribution of microRNAs, encapsulated in bovine milk exosomes, varies among different species of microRNA. Our study also suggests that milk exosomes deliver protein and RNA cargos to the brain, which is consistent with previous reports that genetically engineered murine exosomes deliver Cre recombinase to the brain following nasal administration in mice, and feeding an exosome- and RNA-depleted diet impairs spatial learning and memory in mice^27,32^.

## Results

### Bioavailability and distribution of milk exosomes and protein cargos

**Figure 1:**
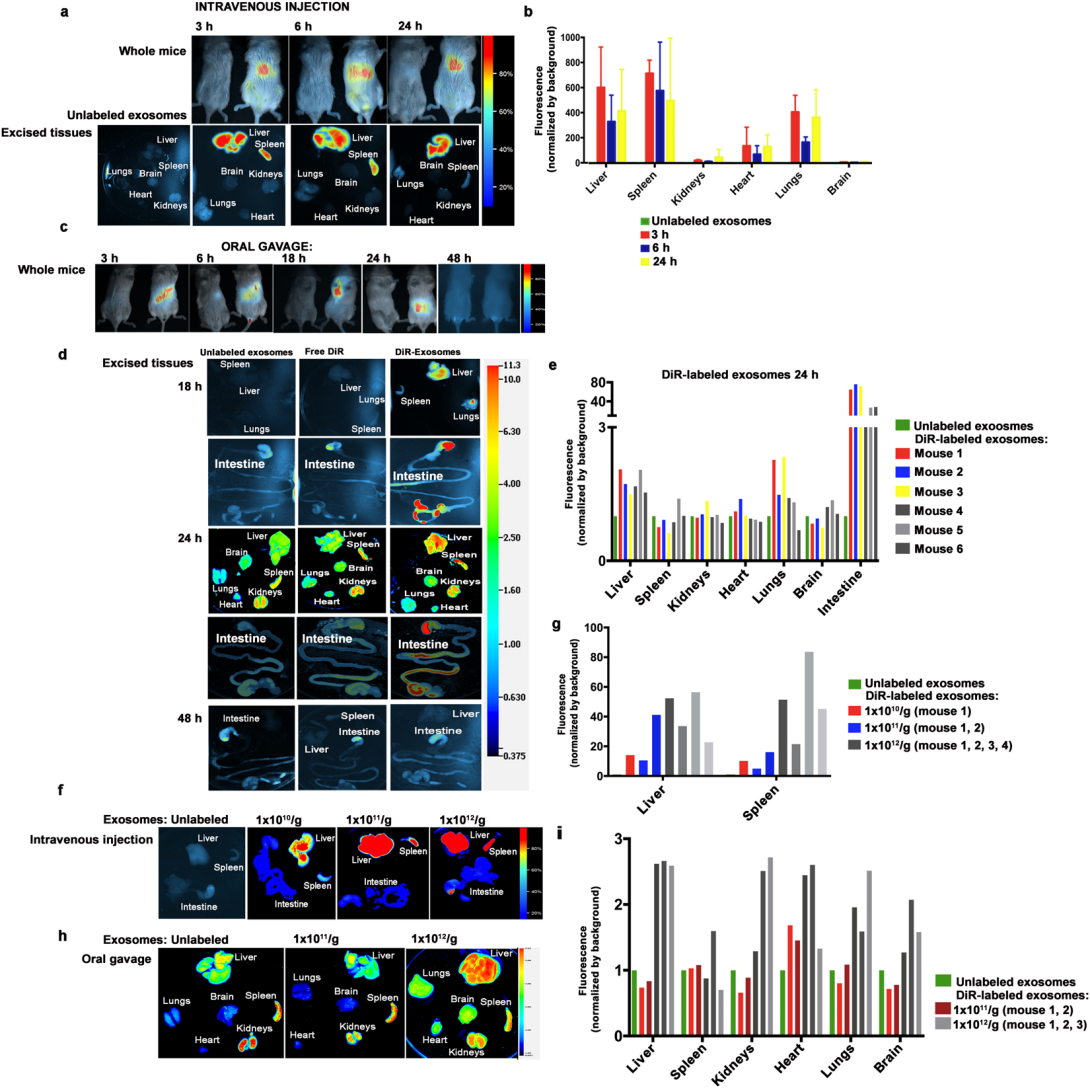
Absorption of bovine milk exosomes in mice. (**a**) DiR-labeled exosomes (1 × 10^12^/g body weight) 3, 6 and 24 hours after intravenous injection in whole Balb/c mice (upper panels) and excised tissues (lower panels). (**b**) Densitometry analysis of fluorescence in excised tissues 3, 6 and 24 hours after intravenous injection of DiR-labeled exosomes. (**c**) Fluorescence in Balb/c mice 3, 6, 18, 24 or 48 hours after oral gavage of DiR-labeled (right mouse) or unlabeled exosomes (left mouse; 1 × 10^12^/g body weight). (**d**) Fluorescence in excised organs of Balb/c mice 3, 6, 18, 24 or 48 hours after oral gavage of free DiR, unlabeled or DiR-labeled exosomes (1 × 10^12^/g body weight). (**e**) Densitometry analysis of fluorescence in excised tissues after oral gavage of unlabeled or DiR-labeled exosomes at 24 hours, normalized for plate background for each mouse analyzed. (**f**) Dose-response analysis of fluorescence in excised murine tissues 24 h after intravenous injection with unlabeled or DiR-labeled exosomes (1 × 10^10^/g, 1 × 10^11^/g, 1 × 10^12^/g body weight). (**g**) Densitometry analysis of excised tissues after intravenous injection of unlabeled or DiR-labeled exosomes for each mouse analyzed (1 × 10^10^/g, 1 × 10^11^/g, 1 × 10^12^/g body weight). (**h**) Dose-response analysis of fluorescence in excised murine tissues 24 h after oral gavage with unlabeled or DiR-labeled exosomes (1 × 10^11^/g, 1 × 10^12^/g body weight) 24 h after oral gavage. (**i**) Densitometry analysis of excised tissues after oral gavage of unlabeled or DiR-labeled exosomes in dose-response experiments. Panels in this figure were assembled from multiple independent images and gels; individual images in the grouped figure are separated by white space.

DiR-labeled bovine milk exosomes administered to adult Balb/c mice through oral gavage yielded a greater fluorescent signal than control animals receiving either free DiR or unlabeled exosomes (see below). The signal produced by free DiR was stronger than the signal produced by unlabeled exosomes; thus, the signals produced by free DiR and DiR-labeled exosomes were compared in all statistical analyses of densitometry data, but the signal produced by unlabeled exosomes served as controls in the bar graphs (see Discussion). The signal produced by DiR-labeled bovine milk exosomes was greater than that produced by free DiR in liver, spleen and, to a lesser extent, in lungs 3 hours after intravenous injection, but was significantly higher only in liver 24 hours after oral gavage in female Balb/c mice (Fig. 1a–e and Supplementary Fig. 1). The tissue accumulation of DiR-labeled milk exosomes was not higher than that of free DiR in male mice (Supplementary Fig. 2). Time course analyses suggested that exosome concentrations in liver and spleen peaked 3 hours after intravenous injection and decreased slightly at subsequent time points (Fig. 1a,b). In contrast, the exosome signal in the liver peaked 24 hours after oral gavage, and no signal was detectable 48 hours after administration (Fig. 1d). A dose-dependent increase in the DiR-exosome signal was observed, suggesting that the minimal doses detectable after intravenous and oral administration were 1 × 10^10^ exosomes/g and 1 × 10^12^ exosomes/g body weight, respectively (Fig. 1f–i and Supplementary Fig. 3). For comparison, our purification protocol yielded about 7 × 10^10^ exosomes/ml of commercial bovine milk.

We attempted to assess the approximate bioavailability of orally versus intravenously administered bovine milk exosomes by comparing the DiR signal pooled from all murine organs following oral gavage and intravenous injection. When DiR signals were compared 24 hours after oral gavage to 3 hours or 24 hours after intravenous injection, the apparent bioavailability was 3.9 ± 2.1% and 5.9 ± 2.6%, respectively. These estimates are substantially lower than those based upon using fluorophore-labeled miR-320a as a marker (see below).

Bioavailability and tissue distribution were altered when exosomes were ultrasonicated or treated with trypsin prior to labeling with DiR (Supplementary Fig. 4). In previous studies, we showed that ultrasonication causes a change in exosomes morphology and substantial loss in RNA cargos, whereas removal of surface proteins by protease treatment causes a loss in exosome uptake by intestinal and vascular endothelial cells^13,15,16,33^. In this study, ultrasonication of exosome caused a loss of bioavailability to levels near the detection limit after oral administration (Supplementary Fig. 4a). Removal of exosomal surface proteins by treatment with trypsin caused a reduced accumulation of exosomes in the liver and homing to lungs and spleen after intravenous injection (Supplementary Fig. 4b). Depletion of macrophages by treating mice with clodronate resulted in an almost exclusive accumulation of exosomes in the liver after oral gavage (Supplementary Fig. 5).

**Figure 2:**
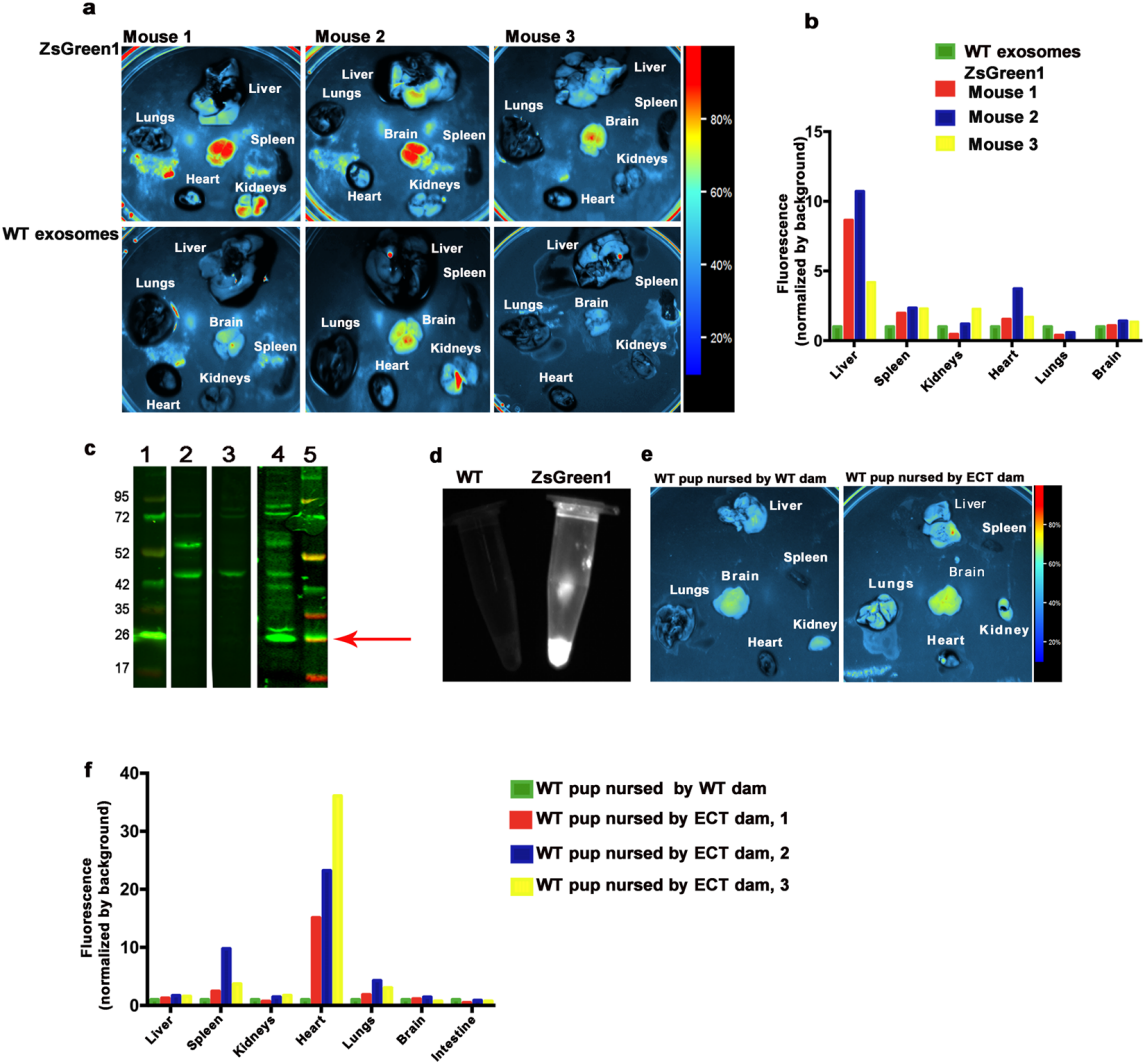
Absorption and distribution of murine and porcine milk exosomes endogenously labeled with fluorescent proteins in mice and pigs. (**a**) Fluorescence signal of porcine ZsGreen1 milk exosomes (upper panel) and WT exosomes (lower panel; 1 × 10^12^/g body weight) in murine tissues excised 3 hours after intravenous injection. (**b**) Densitometry analysis of fluorescence in tissues excised from mice injected with porcine ZsGreen1 or WT milk exosomes. (**c**) Western blot analysis of ZsGreen1 in protein extracts from WT piglets nursed by a transgenic ZsGreen1 sow for 17 days. Lanes: 1, Marker; 2, cerebellum in WT pig; 3, cerebellum in a stillborn (not nursed) WT pig; 4, cerebellum in WT pig nursed by a transgenic sow; and 5, marker. (**d**) Porcine milk exosomes purified from WT (left) and transgenic ZsGreen1 pigs (right). (**e**) CD63/eGFP-labeled exosomes in WT mouse pups nursed by an ECT transgenic dam for 17 days. (**f**) Densitometry analysis of fluorescence in tissues excised from WT pups nursed by an ECT transgenic dam for 17 days compared with tissues excised from WT pups nursed by WT dam for 17 days. Panels in this figure were assembled from multiple independent images and gels; individual images in the grouped figure are separated by white space.

When working with lipophilic fluorophores such as DiR, the transfer of dyes from the labeled complex to other complexes is a concern^34^. Here, we used milk from transgenic pigs and mice to assess the bioavailability and distribution of endogenously-labeled exosomes delivered at physiologically relevant doses through the natural route of suckling. The transgenic pigs and C57BL/6 mice produced milk in which exosomes were endogenously labeled with ZsGreen1 or an enhanced green fluorescent protein (CD63/eGFP), respectively (not shown). While the signal of both green fluorescent proteins was not strong enough to assess bioavailability after a single oral dose (data not shown), both models allowed for the analysis of bioavailability and distribution of milk exosomes after intravenous injection and uptake through suckling. Exosomes endogenously labeled with ZsGreen1 in pig milk were detected in the liver and brain 3 hours after intravenous injection in female Balb/c mice (1 × 10^12^/g body weight) compared with the autofluorescence of organs from mice injected with wild-type (WT) porcine milk exosomes (Fig. 2a,b). Next, WT piglets were nursed by sows secreting ZsGreen1-positive milk exosomes for 17 days. When tissue extracts were probed with anti-ZsGreen1, we detected an immunoreactive protein with a molecular weight of approximately 26 kDa in cerebellum in eight of the 12 piglets that were tested (Fig. 2c); faint signals were also detected in the spleen in some pigs (Supplementary Fig. 6b lanes 5 and 8). The mass of the protein matched the expected molecular weight for ZsGreen^35^, suggesting absence of ZsGreen1 degradation. No band corresponding to the 26-kDa ZsGreen1 signal was detectable in cerebellum samples from WT pigs nursed by a WT dam or in stillborn WT pigs (Fig. 2c and Supplementary Fig. 6). The presence of ZsGreen1 in exosomes was confirmed by using an iBox imaging system (Fig. 2d). A similar pattern of exosomes distribution was observed when WT mouse pups were nursed by CD63/eGFP-positive dams for 17 days (Fig. 2e). The eGFP signal was higher in heart, lungs, kidneys, brain and, to a lesser extent in liver in WT mice pups nursed by CD63/eGFP-positive dams compared to WT pups nursed by WT dams (Fig. 2e,f).

### Bioavailability and distribution of microRNA cargos

**Figure 3:**
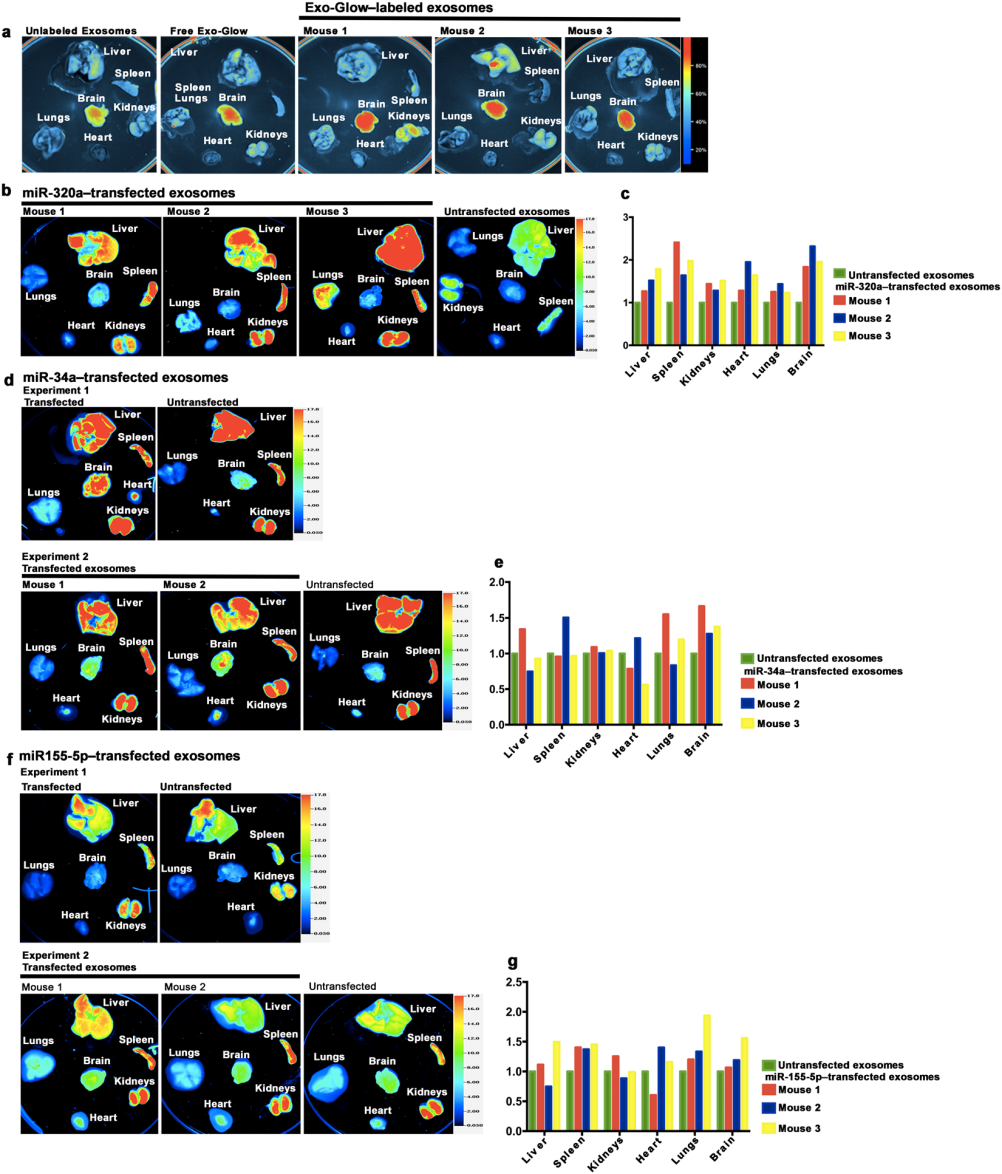
Bioavailability and distribution of fluorophore-labeled RNAs transfected into bovine milk exosomes and administered to mice. (**a**) Fluorescence signal from Exo-Glow-labeled RNA, delivered through oral gavage of bovine milk exosomes (1 × 10^12^/g body weight) in Balb/c mice 12 hours after administration. (**b**) Distribution of synthetic IRDye-labeled miR-320a, transfected into milk exosomes (1 × 10^12^/g body weight) 6 hours after delivery of exosomes by oral gavage in Balb/c mice. (**c**) Densitometry analysis of miR-320a distribution shown in panel b. (**d**) Distribution of synthetic IRDye-labeled miR-34a, transfected into milk exosomes (1 × 10^12^/g body weight) 6 hours after delivery of exosomes by oral gavage in Balb/c mice. Data from two experiments are shown. (**e**) Densitometry analysis of miR-34a distribution shown in panel d. (**f**) Distribution of synthetic IRDye-labeled miR-155-5p, transfected into milk exosomes (1 × 10^12^/g body weight) 6 hours after delivery of exosomes by oral gavage in Balb/c mice (n = 3). (**g**) Densitometry analysis of miR-155-5p distribution shown in panel f. Panels in this figure were assembled from multiple independent images; individual images in the grouped figure are separated by white space.

Distinct species of microRNAs in bovine milk exosomes showed unique profiles of bioavailability and distribution, and the distribution was dissimilar compared to that of exosomes in Balb/c mice. As a first line of evidence, the entire pool of single-stranded RNAs in bovine milk exosomes was labeled using Exo-Glow Red. The majority of labeled RNA localized to brain after intravenous injection with peak levels occurring 2 hours after injection compared to controls injected with unlabeled exosomes (Supplementary Fig. 7). In contrast, after oral gavage the majority of the labeled RNA localized to brain, kidneys and liver with peak levels occurring 12 hours after administration (Fig. 3a). Two controls, free Exo-Glow and unlabeled exosomes, produced a signal weaker than that produced by exosomal RNAs labeled with Exo-Glow following oral gavage.

Next, we devised a novel protocol and transfected bovine milk exosomes with synthetic IRDye-labeled microRNAs; exosomes treated IRDye-labeled microRNAs in the absence of transfection reagent were administered to controls. The four microRNAs that were tested displayed unique patterns of tissue distribution. MiR-320a accumulated primarily in liver with small amounts being detectable in kidneys, lungs and spleen 3 hours after intravenous injection (Supplementary Fig. 8); in contrast, miR-320a accumulated primarily in liver, spleen and kidneys 6 hours after oral gavage, and the signals were greater than those produced by transfection reagent-free controls (Fig. 3b,c). IRDye is a near-infrared dye, which explains the strong signals produced by IRDye-labeled microRNAs compared with some of other labels used in our studies. When the fluorescent signal was compared after intravenous and oral administration, the apparent bioavailability of miR-320a was 25.4 ± 8.7%, based on the comparison of hepatic densitometry data 3 hours after intravenous injection and 6 hours after oral gavage. MiR-34a and miR-155-5p accumulated primarily in the brain and spleen, respectively, following oral gavage (Fig. 3d–g).

**Figure 4:**
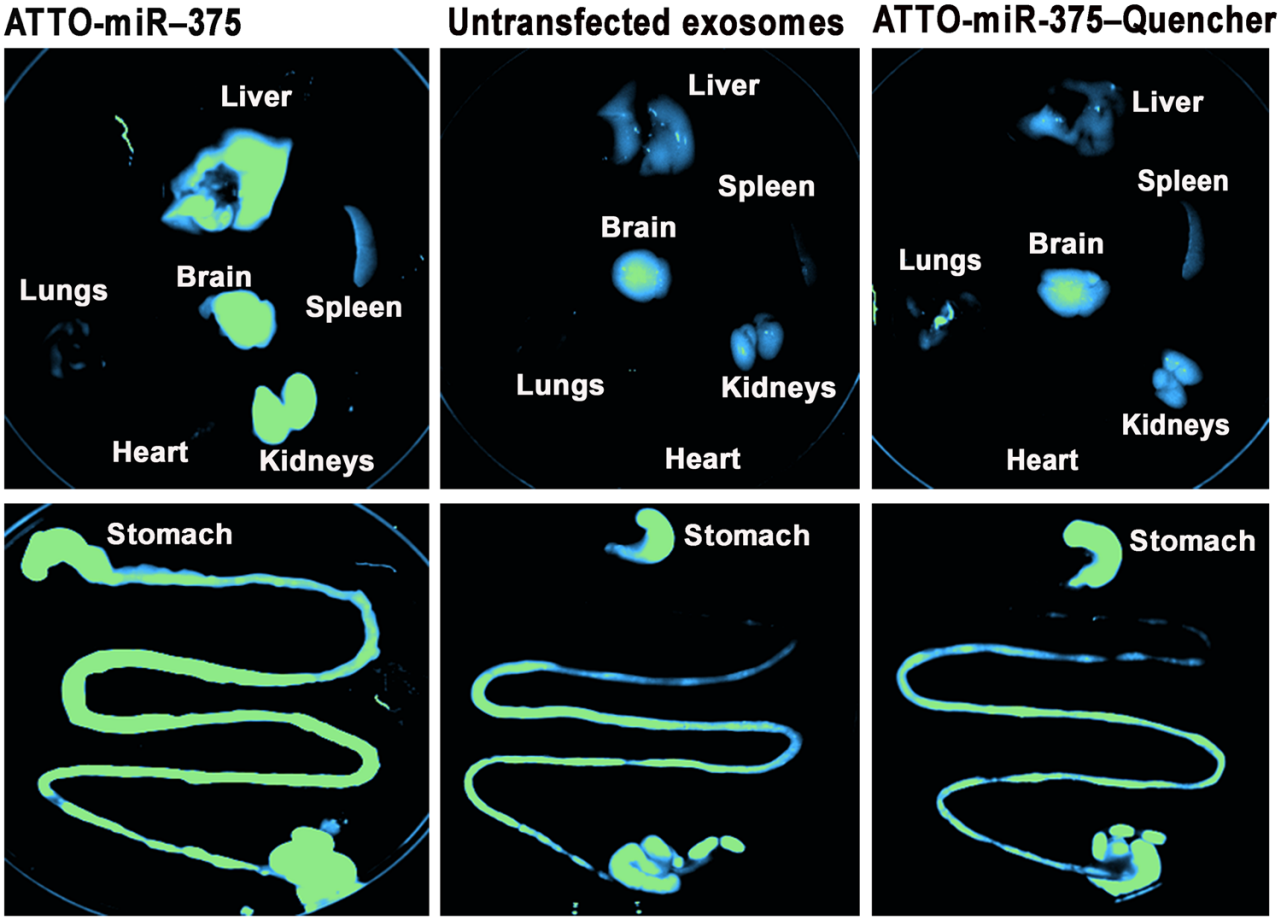
Assessment of microRNA degradation using a dual-label protocol in mice. Distribution of synthetic 5ATTO633N-miR-375 and the corresponding quencher (3IAbRQSp) transfected into milk exosomes (1 × 10^12^/g of body weight) 6 hours after delivery of exosomes by oral gavage in Balb/c mice. The transfection with 5ATTO633N only was used as a control of transfection efficiency and labeling stability. Panels in this figure were assembled from multiple independent images; individual images in the grouped figure are separated by white space.

We used miR-375 to demonstrate that fluorophore labels remained attached to synthetic microRNAs after administration to mice. For this purpose, we synthesized miR-375 covalently labeled with both a fluorophore (5ATTO633N) and the corresponding quencher (3IAbRQSp). The intact microRNA did not emit fluorescence, but produced a signal when the quencher was removed from the fluorophore by treatment with RNase *in vitro* (Supplementary Fig. 9). When miR-375, conjugated to fluorophore and quencher, was administered orally to mice, only a minimal signal was detected in heart and spleen. In contrast, when synthetic 5ATTO633N-labeled miR-375 (no quencher) was administered orally to mice, the signal localized primarily to the intestines, with minor signal being detectable in kidneys, brain and liver (Fig. 4).

We determined whether bovine milk exosomes deliver bioactive microRNAs in quantities sufficient to rescue *Drosha* knockout mice, which cannot synthesize mature microRNAs except for a few *Drosha*-independent microRNAs^36^. Mice were fed a bovine milk exosome and RNA-sufficient (ERS) diet or an exosome and RNA-depleted (ERD) diet and *Drosha* was knocked out ubiquitously by tamoxifen administration at age 21 days; both diets were based on the AIN-93G formula and were identical for all compounds other than exosomes and RNAs. Note that the ERD diet was prepared by using ultrasonicated milk as a supplement and ultrasonicated exosomes are not bioavailable (see above). The lifespan of mice fed the ERS diet (8.3 ± 0.5 d) was not significantly different from that of mice fed the ERD diet (7.2 ± 0.4 d; *p* > 0.05; n = 10 per group).

## Discussion

This report represents a major advance in our understanding of the bioavailability and distribution of dietary (milk) exosomes and their cargos within and beyond species boundaries. Key advances include the following discoveries. First, our findings suggest that bovine milk exosomes are bioavailable and accumulate primarily in the liver and, to a lesser extent, in the spleen of mice. This paper and a previous report suggest that resident macrophages in liver and spleen are responsible for clearing foreign exosomes administered to mice^31^. Our findings are only partially consistent with another previous report suggesting that bovine milk exosomes distribute widely among murine tissues^19^. The different findings in that previous report and our paper could be attributed to the fact that the previous report did not use controls (e.g., free fluorophore or unlabeled exosomes) and used oral doses 4-fold higher than those used in our paper^19^. The accurate identification of exosome target tissues is an important topic, because of efforts to use milk exosomes as vehicles for drug delivery^19,20^. Based on this paper, we propose that bovine exosomes might represent an excellent vehicle for delivering drugs to macrophages and that homing signals within milk exosomes need to be identified that allow targeting drug-loaded milk exosomes to cell lineages other than macrophages. In this context, possible effects of sex on the metabolism of bovine milk exosomes need to be explored since florescent signal was reduced in organs of male mice. The choice of controls is an important consideration in exosome labeling studies. We propose that the administration of free DiR produces an artificially high background signal, based upon the following rationale. The dose of free DiR administered to mice is the same as the amount of DiR used for exosome labeling. However, not all the DiR used in labeling studies binds to exosomes and the unbound fraction was removed prior to the administration of DiR-labeled exosomes to mice. This being said, we erred on the side of caution and used the signal produced by free DiR in statistical comparisons with the signal produced by DiR-labeled exosomes. All tracers used in this study, except for DiR, consistently suggest that milk exosomes and their cargos accumulate in brain, which is consistent with the delivery of Cre recombinase to the brain by exosomes in a previous report^32^. We propose that the bioavailability and distribution patterns observed for Exo-Glow, fusion proteins and fluorophore-labeled microRNAs represent a more accurate picture of exosome bioavailability and distribution than the pattern observed for the less stable DiR-label^34^.

Second, this is the first report to use exosomes endogenously labeled with fluorescent proteins in milk from transgenic pigs and mice. Our studies in these animals suggest that milk exosomes, delivered within species boundaries through the natural route of suckling, distributes differently than foreign milk exosomes. Autologous milk exosomes can be detected in most tissues studied, including the brain. The delivery of exosomes and their protein cargos (ZsGreen1 and CD63/eGFP) is consistent with a previous study suggesting that exosomes endogenously loaded with Cre recombinase deliver the cargo to various brain regions in Cre reporter mice^32^. The accumulation of exosomes and their cargos in the brain is of particular interest in the context of ongoing studies in our laboratory, suggesting that dietary depletion of milk exosomes and their cargos causes a loss of spatial learning and memory in mice^27^. The loss in spatial learning and memory might be due to the aberrant metabolism of purines that has been observed in humans and infants as well as mice fed exosome and RNA cargo-depleted diets^26^; spatial learning and memory depends on purinergic receptor signaling^26,37^. These studies in transgenic pigs and mice also added a new dimension to previous work by suggesting that the consumption of physiological amounts of exosomes through the natural route of suckling causes a detectable accumulation of exosomes in the tissues of piglets and mouse pups. We anticipate that these observations will be an incentive for conducting studies of exosomes and their cargos in human milk and their roles in infant nutrition.

Third, this report provides strong evidence that the milk exosome signal in tissues is not an artifact caused by tracking fluorophores detached from exosomes. While the transfer of lipophilic labels such as DiR from exosomes to proteins and lipoproteins is possible^34^, our green fluorescent proteins in porcine milk and presumably murine milk were stable. Label stability was further confirmed using synthetic miR-375 covalently modified with fluorophore and quencher and administered to mice by using bovine milk exosomes as vehicle. These are significant observations in the context of dietary exosomes and cargos, which has been a contested field of study^17,38–42^.

Fourth, this is the first report that analyzes the biodistribution of different microRNA cargos in bovine milk exosomes. The bulk of endogenous RNAs from exosomes accumulates in the spleen and brain, which is distinct from the distribution of the exosomes themselves. We speculate that microRNA cargos may be transferred from foreign milk exosomes to endogenous exosomes for delivery to the brain, but this theory remains to be tested. When we increased the resolution of the distribution studies by using synthetic fluorophore-labeled microRNAs, as opposed to using the universal Exo-Glow label, a unique pattern of RNA distribution became apparent. Two examples are worth highlighting. MiR-34a has been implicated in spatial cognitive function, hippocampal neurogenesis and brain aging^43,44^. Synthetic miR-34a, transfected into bovine milk exosomes, accumulated primarily in the brain. We speculate that the distribution of microRNAs depends not only on the abundance of complementary mRNAs in tissues, analogous to the principle used in microRNA sponges^45^, but also on the sequence motif of miRNAs that can bind to ribonucleoproteins and facilitate miRNA sorting and packaging into exosomes^46^.

Fifth, the apparent bioavailability of DiR-labeled exosomes, but not RNA cargos is low. That said, the methods used to assess the bioavailability of DiR-labeled exosomes depended on the use of single time points in single tissues and therefore may not represent the true bioavailability. The pharmacokinetics of compounds may greatly depend on the route of administration^47^. Loss of label in the intestine might be another explanation, particularly when considering that the apparent bioavailability of synthetic miR-320a approached 25%. In addition, it is plausible to propose that the bioavailability of milk exosomes within species boundaries is higher than the bioavailability of exosomes beyond species boundaries. Along the lines of bioavailability, feeding the ERS diet did not significantly extend the lifespan of *Drosha* knockout mice compared to mice fed the ERD diet. Failure to rescue the mice is probably due to inadequate quality and relatively low bioavailability of milk microRNAs. For example, about 400 microRNAs have been identified in bovine milk, whereas about 2000 mature murine microRNAs have been identified. Thus, the microRNAs delivered by bovine milk likely lack some microRNAs essential for mice^48,49^.

The findings reported here are guiding ongoing studies in our laboratory. We are in the process of examining the phenotypes of dietary depletion of exosomes and their cargos. As described above, some phenotypes have already emerged and include aberrant metabolism of purines, impaired spatial learning and memory, regulation of DNA methyltransferase 1, as well as impaired resistance to seizures caused by kainic acid^21,26,27^. Based on the observation that a fraction of dietary exosomes and RNAs are not absorbed and enter the large intestine, it will be important to understand the biological effects of milk exosomes and cargos on microbial communities in the gut. Finally, microRNAs are not the only species of RNA in milk exosomes. It will be worthwhile to explore the biological effects of exosome-dependent delivery of RNAs other than microRNAs beyond and within species boundaries. For example, single-stranded RNAs are ligands for Toll-like receptors, which is consistent with immunomodulatory effects of milk in infants^50,51^.

## Methods

### Animals

All animal procedures (animal handling, experimental procedure, anesthesia and euthanasia) were conducted in accordance with the University of Nebraska-Lincoln’s Institutional Animal Care and Use Committee and approved by the Institutional Animal Care Program (protocol 1229). Studies were conducted in female Balb/c mice (Jackson Laboratory, stock number 000651), ages 8 to 20 weeks, unless noted otherwise. Exosome and Cargo Tracking (ECT) mice were developed in our laboratory and express an exosome marker protein (CD63) fused to eGFP. The presence of the CD63/eGFP gene was confirmed by PCR (Table 1); expression of the transgene was confirmed by imaging green fluorescence (excitation 455–495 nm, emission 503–523 nm). Homozygous *Drosha* knockout mice^52^, a gift by Dr. Tatsuya Kobayashi (Massachusetts General Hospital), were mated to tamoxifen-inducible R26CreER mice (Jackson Labs, stock number: 004847). Homozygous tamoxifen-inducible *Drosha* knockout mice were identified by PCR (Table 1). Transgenic pigs with ubiquitous ZsGreen1 expression were developed as described previously^53^.

**Table 1:** PCR primers.

Primers	Gene
5′-GCAGAAAGTCTCCCACTCCTAACCTTC-3′ (F)	*Drosha*
5′-CCAGGGGAAATTAAACGAGACTCC-3′ (R)	
5′-AAGGGAGCTGCAGTGGAGTA-3′ (F)	*Cre*
5′-CCGAAAATCTGTGGGAAGTC-3′ (R)	
5′-TCTTGCGAACCTCATCACTC-3′ (R)	
5′-GCAAGAGGTGCGGAAGATTA-3′ (F)	*CD63/eGFP*
5′-GGATGGCGAAGCTAAGATCAA-3′ (R)	

### Exosome isolation and authentication

Fat-free pasteurized (skim) bovine milk was obtained from a local grocery store; for pasteurization the milk was heated at 71.6 °C for 15 seconds. We chose fat-free pasteurized milk rather than ultra-heat treated milk, because ultra-heat treatment causes a loss of milk microRNAs and presumably exosomes^54^. Exosomes were isolated by ultracentrifugation as described previously, with minor modifications^15,18^. The combined use of fat-free milk and exosome purification by ultracentrifugation minimized sample contamination with fat globules, which may also contain microRNAs^55^. Exosomes were authenticated by transmission electron microscopy, nanoparticle tracking analysis (NanoSight NS300, Malvern, Inc.), and western blot analysis following the guidelines by the International Society for Extracellular Vesicles (Supplementary Fig. 10)^16,56^. Exosomes were suspended in sterile phosphate-buffered saline (PBS) and stored at −80 °C for up to 120 days^57^. Porcine milk exosomes were isolated as described above; the presence of ZsGreen1 was confirmed by western blot analysis using anti-ZsGreen1 (cat #: 632598; Clontech) and by assessing ZsGreen1 fluorescence in an iBox small animal imaging system (UVP LLC).

Some experiments were conducted in mice depleted of macrophages by treatment with 150 μl clodronate liposomes (clophosome-A^TM^, FormuMax, Inc.) administered intraperitoneally 24 hours before exosome administration^31^; controls were treated with PBS (Supplementary Fig. 5). Selected experiments were conducted using exosomes depleted of surface proteins by treatment with 50 µg/ml trypsin at 37 °C for 30 min, followed by a wash with sterile PBS (120,000 × *g* for 1.5 h) and resuspension in sterile PBS. Experiments of depletion of exosomes from milk were performed ultrasonicating milk for 1 hour and followed by incubation at 37 °C for 1 hour before exosome isolation (Branson 5800; 47 kHz frequency and 185 Watt peak power).

### Labeling of exosomes and RNAs with synthetic fluorophores

Exosomes were labeled with the lipophilic fluorophore 1,1-dioctadecyl-3,3,3,3-tetrametylindotricarbocyanine iodide (DiR) as described previously^58^. The signal of DiR-labeled exosomes in murine tissues was compared to those produced by an equal number of exosomes and free DiR. Only when the signal of DiR-labeled exosomes was significantly greater than signals produced by free DiR, tissues were considered potential sites of exosome accumulation. This approach represents a conservative method to identifying exosome accumulation sites (see Discussion). If images were collected in independent sessions, the signal intensity was normalized across plates by using the signal intensities in tissue-free sections of the plates as reference. RNAs endogenous to bovine milk exosomes were labeled using Acridine Orange chemistry (Exo-Glow Red kit, System Biosciences, Inc.) according to the manufacturer’s instructions; the stain allows for tracking of the entire pool of single-stranded RNAs. Some exosomes were transfected with synthetic miR-375, labeled with a fluorophore (5ATTO633N,excitation 635, emission 653 nm) and the corresponding quencher 3IAbRQSp (ITDNA, Inc), or IRDye-labeled miR-320a, miR-155 or miR-34a (IDTDNA, Inc). For transfection, 1 × 10^12^ exosomes were incubated with 120 pmoles microRNA), 0.5 mM calcium chloride and 40% ethanol in a volume of 1 ml for 15 minutes (personal communication by Drs T. Ochiya and K. Othsuka K. and Ochiya T., Division of Molecular and Cellular Medicine, National Cancer Center Research Institute, 5-1-1, Tsukiji, Chuo-ku, Tokyo, Japan). Free dyes and extra-exosomal synthetic microRNAs were removed by two PBS washes (120,000 × *g* for 60 min), and 1 × 10^12^ exosomes were administered per g body weight in a volume of less than 150 μl. Untransfected controls were prepared by omitting calcium chloride and ethanol. All exosome preparations were utilized within 2 hours.

### Rescue experiments

Tamoxifen-inducible *Drosha* knockout mice were fed exosome and RNA-depleted (ERD) diets or exosome and RNA-sufficient (ERS) diets starting at 3 weeks of age^13^. Unbiased randomization was achieved by numbering the mice and assigning numbers to treatment groups using a blinded design^59^. At 3 weeks of age, tamoxifen was administered intraperitoneally (~80 mg/kg body weight), followed by a second injection 48 hours later^60^. Mice were monitored for survival at 12-hour intervals.

### Imaging experiments

Fluorescence intensities were assessed at timed intervals for up to 48 hours after exosome administration using an iBox small animal imaging system and customized wavelength filters (UVP, LLC) for live animals or a LI-COR Odyssey^®^ imaging system (LI-COR Biosciences) for excised organs. Imaging experiments were conducted primarily in dissected tissues that were rinsed with cold PBS to remove blood; some experiments were conducted in live mice anesthetized with isoflurane. DiR-labeled exosomes and IRDye-labeled microRNAs were assessed using 748 nm for excitation and 780 nm for emission. Dissected tissues were rinsed in cold PBS and fluorescence was detected by using LI-COR Odyssey^®^ imaging system within 1 hour post-mortem; densitometry analysis was performed with LI-COR Image Studio Lite software. Acridine Orange Exo-Glow Red-labeled RNA was detected in dissected tissues by using iBox small animal imaging system with customized wavelength filters (460 nm excitation, 650 nm emission) and densitometry analysis was performed with VisionWorks^®^ LS software (UVP, LLC.). The distribution of endogenously labeled ECT exosomes in murine tissues was analyzed by using an iBox small animal imaging system and VisionWorks^®^ LS software.

Since ECT dams are hemizygous, matings with WT male mice result in litters that are 50% WT and 50% hemizygous for the CD63/eGFP transgene. Litters born to ECT dams were nursed for 17 days; pups were euthanized and WT pups were identified by genotyping. The accumulation of CD63/eGFP-labeled exosomes was analyzed in excised tissues, such as liver, lungs, brain, kidneys, spleen, heart and the intestine by using an iBox small animal imaging system (UVP LLC.). WT pups nursed by WT dams were used as negative controls. The experimental design was the same in ZsGreen1 pigs. WT piglets were nursed by transgenic ZsGreen1 sows for 17 days and tissues were collected and analyzed by western blot analysis using anti-ZsGreen1. WT piglets nursed by WT sows and stillborn WT piglets were used as negative controls. For studies of ZsGreen1-labeled exosomes in mice, porcine milk was manually collected and frozen at −20 °C until use.

### Statistics

Statistical analysis was performed using Prism 7.0 (GraphPad Software Inc.) by using the *t*-test for *p*-values. All results are expressed as the mean ± standard deviation (SD).

## Electronic supplementary material

Supplementary Figures

## References

[1] Yanez-Mo M (2015). Biological properties of extracellular vesicles and their physiological functions. J. Extracell. Vesicles.

[2] Abels ER, Breakefield XO (2016). Introduction to Extracellular Vesicles: Biogenesis, RNA Cargo Selection, Content, Release, and Uptake. Cell. Mol. Neurobiol..

[3] Raposo G, Stoorvogel W (2013). Extracellular vesicles: exosomes, microvesicles, and friends. J. Cell Biol..

[4] Chong MM, Rasmussen JP, Rudensky AY, Littman DR (2008). The RNAseIII enzyme Drosha is critical in T cells for preventing lethal inflammatory disease. J. Exp. Med..

[5] Friedman RC, Farh KK, Burge CB, Bartel DP (2009). Most mammalian mRNAs are conserved targets of microRNAs. Genome Res..

[6] Vojtech L (2014). Exosomes in human semen carry a distinctive repertoire of small non-coding RNAs with potential regulatory functions. Nucleic Acids Res..

[7] Danielson KM, Das S (2014). Extracellular Vesicles in Heart Disease: Excitement for the Future?. Exosomes Microvesicles.

[8] Koeck ES (2014). Adipocyte exosomes induce transforming growth factor beta pathway dysregulation in hepatocytes: a novel paradigm for obesity-related liver disease. J. Surg. Res..

[9] Thomou T (2017). Adipose-derived circulating miRNAs regulate gene expression in other tissues. Nature.

[10] Bellingham SA, Guo BB, Coleman BM, Hill AF (2012). Exosomes: vehicles for the transfer of toxic proteins associated with neurodegenerative diseases?. Front. Physiol..

[11] Vella, L. J., Hill, A. F. & Cheng, L. Focus on Extracellular Vesicles: Exosomes and Their Role in Protein Trafficking and Biomarker Potential in Alzheimer’s and Parkinson’s Disease. *Int*. *J*. *Mol*. *Sci*. **17**, 10.3390/ijms17020173 (2016). 10.3390/ijms17020173 PMC4783907 26861304

[12] Andras IE, Toborek M (2016). Extracellular vesicles of the blood-brain barrier. Tissue Barriers.

[13] Baier SR, Nguyen C, Xie F, Wood JR, Zempleni J (2014). MicroRNAs are absorbed in biologically meaningful amounts from nutritionally relevant doses of cow’s milk and affect gene expression in peripheral blood mononuclear cells, HEK-293 kidney cell cultures, and mouse livers. J. Nutr..

[14] Shu J, Chiang K, Zempleni J, Cui J (2015). Computational characterization of exogenous microRNAs that can be transferred into human circulation. Plos One.

[15] Wolf T, Baier SR, Zempleni J (2015). The intestinal transport of bovine milk exosomes is mediated by endocytosis in human colon carcinoma caco-2 cells and rat small intestinal IEC-6 cells. J. Nutr..

[16] Kusuma Jati R (2016). Human vascular endothelial cells transport foreign exosomes from cow’s milk by endocytosis. Am. J. Physiol. Cell Physiol..

[17] Wang L, Sadri M, Giraud D, Zempleni J (2018). RNase H2-dependent polymerase chain reaction and elimination of confounders in sample collection, storage, and analysis strengthen evidence that microRNAs in bovine nilk are bioavailable in humans. J. Nutr..

[18] Izumi H (2015). Bovine milk exosomes contain microRNA and mRNA and are taken up by human macrophages. J. Dairy Sci..

[19] Munagala R, Aqil F, Jeyabalan J, Gupta RC (2016). Bovine milk-derived exosomes for drug delivery. Cancer Lett..

[20] Vashisht M, Rani P, Onteru SK, Singh D (2017). Curcumin Encapsulated in Milk Exosomes Resists Human Digestion and Possesses Enhanced Intestinal Permeability *in Vitro*. Appl. Biochem. Biotechnol..

[21] Golan-Gerstl, R. *et al*. Characterization and biological function of milk-derived miRNAs. *Mol*. *Nutr*. *Food Res*. **61**, 10.1002/mnfr.201700009 (2017). 10.1002/mnfr.201700009 28643865

[22] Chen T (2016). Porcine milk-derived exosomes promote proliferation of intestinal epithelial cells. Sci. Rep..

[23] Izumi H (2012). Bovine milk contains microRNA and messenger RNA that are stable under degradative conditions. J. Dairy Sci..

[24] Benmoussa A (2016). Commercial dairy cow milk microRNAs resist digestion under simulated gastrointestinal tract conditions. J. Nutr..

[25] Liao Y, Du X, Li J, Lonnerdal B (2017). Human milk exosomes and their microRNAs survive digestion *in vitro* and are taken up by human intestinal cells. Mol. Nutr. Food Res..

[26] Aguilar-Lozano, A. *et al*. Depletion of dietary microRNAs from cow’s milk causes an increase of purine metabolites in human body fluids and mouse livers. *FASEB J*. 30 (supplement 1), 127.121, [peer-reviewed meeting abstract] (2016).

[27] Mutai, E., Zhou, F. & Zempleni, J. Depletion of dietary bovine milk exosomes impairs sensorimotor gating and spatial learning in C57BL/6 mice. *FASEB J*. 31, 150.154 [peer-reviewed meeting abstract] (2017).

[28] National Cancer Institute. NCI dictionary of cancer terms. http://www.cancer.gov/dictionary?cdrid=703278 (accessed 7/6/2014).

[29] Song Y (2013). Whole milk intake is associated with prostate cancer-specific mortality among U.S. male physicians. J. Nutr..

[30] Tat D (2018). Milk and other dairy foods in relation to prostate cancer recurrence: Data from the cancer of the prostate strategic urologic research endeavor (CaPSURE). Prostate.

[31] Imai T (2015). Macrophage-dependent clearance of systemically administered B16BL6-derived exosomes from the blood circulation in mice. J. Extracell. Vesicles.

[32] Sterzenbach U (2017). Engineered Exosomes as Vehicles for Biologically Active Proteins. Mol. Ther..

[33] Sukreet, S., Zhang, H., Adamec, J., Cui, J. & Zempleni, J. Identification of glycoproteins on the surface of bovine milk exosomes and intestinal cells that facilitate exosome uptake in human colon carcinoma Caco-2 cells. *FASEB J*. 31, 646.625, [peer-reviewed meeting abstract] (2017).

[34] Takov K, Yellon DM, Davidson SM (2017). Confounding factors in vesicle uptake studies using fluorescent lipophilic membrane dyes. J. Extracell. Vesicles.

[35] Matz MV (1999). Fluorescent proteins from nonbioluminescent Anthozoa species. Nat. Biotechnol..

[36] Kim YK, Kim B, Kim VN (2016). Re-evaluation of the roles of DROSHA, Export in 5, and DICER in microRNA biogenesis. Proc. Natl. Acad. Sci. USA.

[37] Duster R, Prickaerts J, Blokland A (2014). Purinergic signaling and hippocampal long-term potentiation. Curr. Neuropharmacol..

[38] Title AC, Denzler R, Stoffel M (2015). Uptake and function studies of maternal milk-derived microRNAs. J. Biol. Chem..

[39] Laubier J, Castille J, Le Guillou S, Le Provost F (2015). No effect of an elevated miR-30b level in mouse milk on its level in pup tissues. RNA Biol..

[40] Zempleni J, Baier SR, Hirschi K (2015). Diet-responsive microRNAs are likely exogenous. J. Biol. Chem..

[41] Zempleni J (2017). Biological activities of extracellular vesicles and their cargos from bovine and human milk in humans and implications for infants. J. Nutr..

[42] Hirschi KD (2017). Navigating dietary small RNAs. Genes Nutr..

[43] Li X, Khanna A, Li N, Wang E (2011). Circulatory miR34a as an RNAbased, noninvasive biomarker for brain aging. Aging (Albany NY).

[44] Zhang QJ, Li J, Zhang SY (2017). Effects of TRPM7/miR-34a gene silencing on spatial cognitive function and hippocampal neurogenesis in mice with Type 1 Diabetes mellitus. Mol. Neurobiol..

[45] Ebert MS, Neilson JR, Sharp PA (2007). MicroRNA sponges: competitive inhibitors of small RNAs in mammalian cells. Nat. Methods.

[46] Shurtleff MJ, Temoche-Diaz MM, Karfilis KV, Ri S, Schekman R (2016). Y-box protein 1 is required to sort microRNAs into exosomes in cells and in a cell-free reaction. eLife.

[47] van Duijkeren E (1994). A comparative study of the pharmacokinetics of intravenous and oral trimethoprim/sulfadiazine formulations in the horse. J. Vet. Pharmacol. Ther..

[48] Sun J (2015). MicroRNA expression profiles of bovine milk exosomes in response to *Staphylococcus aureus* infection. BMC Genomics.

[49] Kozomara A, Griffiths-Jones S (2014). miRBase: annotating high confidence microRNAs using deep sequencing data. Nucleic Acids Res..

[50] Admyre C (2007). Exosomes with immune modulatory features are present in human breast milk. J. Immunol..

[51] Waltenbaugh, C., Doan T., Melvold R. & Viselli S. Immunology. (Wolters Kluwer Health/Lippincott Williams & Wilkins, 2008).

[52] Chong MM (2010). Canonical and alternate functions of the microRNA biogenesis machinery. Genes Dev..

[53] Desaulniers AT, Cederberg RA, Mills GA, Lents CA, White BR (2017). Production of a gonadotropin-releasing hormone 2 receptor knockdown (GNRHR2 KD) swine line. Transgenic Res..

[54] Kirchner B, Pfaffl MW, Dumpler J, von Mutius E, Ege M (2016). J. microRNA in native and processed cow’s milk and its implication for the farm milk effect on asthma. J. Allergy Clin. Immunol..

[55] Alsaweed M, Lai CT, Hartmann PE, Geddes DT, Kakulas F (2016). Human milk miRNAs primarily originate from the mammary gland resulting in unique miRNA profiles of fractionated milk. Sci. Rep..

[56] Lotvall J (2014). Minimal experimental requirements for definition of extracellular vesicles and their functions: a position statement from the International Society for Extracellular Vesicles. J. Extracell. Vesicles.

[57] Wu Y, Deng W, Klinke DJ (2015). Exosomes: improved methods to characterize their morphology, RNA content, and surface protein biomarkers. Analyst.

[58] Wiklander OP (2015). Extracellular vesicle *in vivo* biodistribution is determined by cell source, route of administration and targeting. J. Extracell. Vesicles.

[59] Couzin-Frankel J (2013). When mice mislead. Science.

[60] Andersson KB, Winer LH, Mork HK, Molkentin JD, Jaisser F (2010). Tamoxifen administration routes and dosage for inducible Cre-mediated gene disruption in mouse hearts. Transgenic Res..

