# Supplementary material for: Milk exosomes are bioavailable and distinct microRNA cargos have unique tissue distribution patterns
Source: Sci Rep. 2018 Jul 27;8:11321. doi: 10.1038/s41598-018-29780-1 (PMC6063888; doi:10.1038/s41598-018-29780-1)
Supplement: Supplementary file 1 — Supplementary Figures [file 41598_2018_29780_MOESM1_ESM.pdf]

Supplementary Information for the manuscript Milk exosomes are bioavailable and distinct microRNA cargos have unique tissue distribution patterns

Sonia Manca[1], Bijaya Upadhyaya[1], Ezra Mutai[1], Amy T. Desaulniers[2], Rebecca A. Cederberg[2], Brett R. White[2] & Janos Zempleni[1,*]

[1]Department of Nutrition and Health Sciences, University of Nebraska-Lincoln, 316 Leverton Hall, Lincoln, NE 68583-0806, USA. [2]Department of Animal Science, University of Nebraska-Lincoln, A224j Animal Science Building, 3940 Fair Street, Lincoln, NE, 68583-0908, USA.

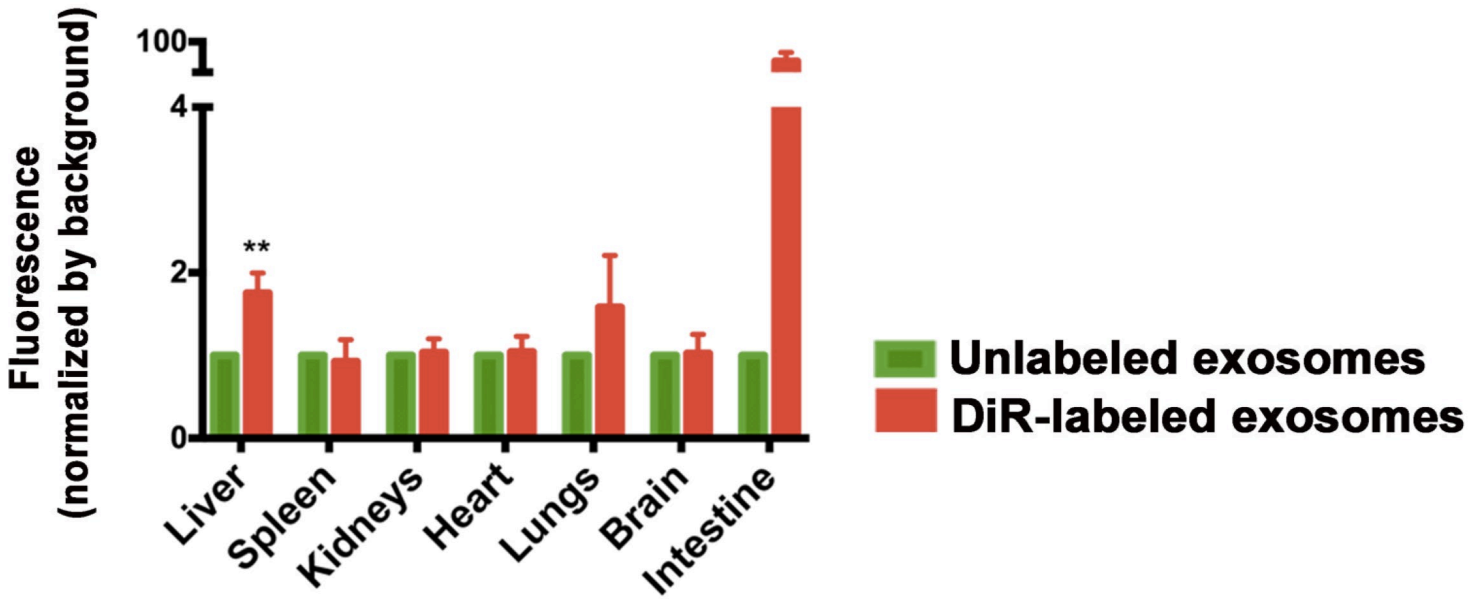

Supplementary Fig. 1 Mean of densitometry analyses shown in Fig. 1e. Mean fluorescence in excised tissues after oral gavage of unlabeled exosomes and DiR-labeled exosomes 24 hours after administration, normalized for plate background (unpaired *t*-test $^{**}p < 0.01$ DiR-labeled exosomes *vs* free DiR, n = 6).

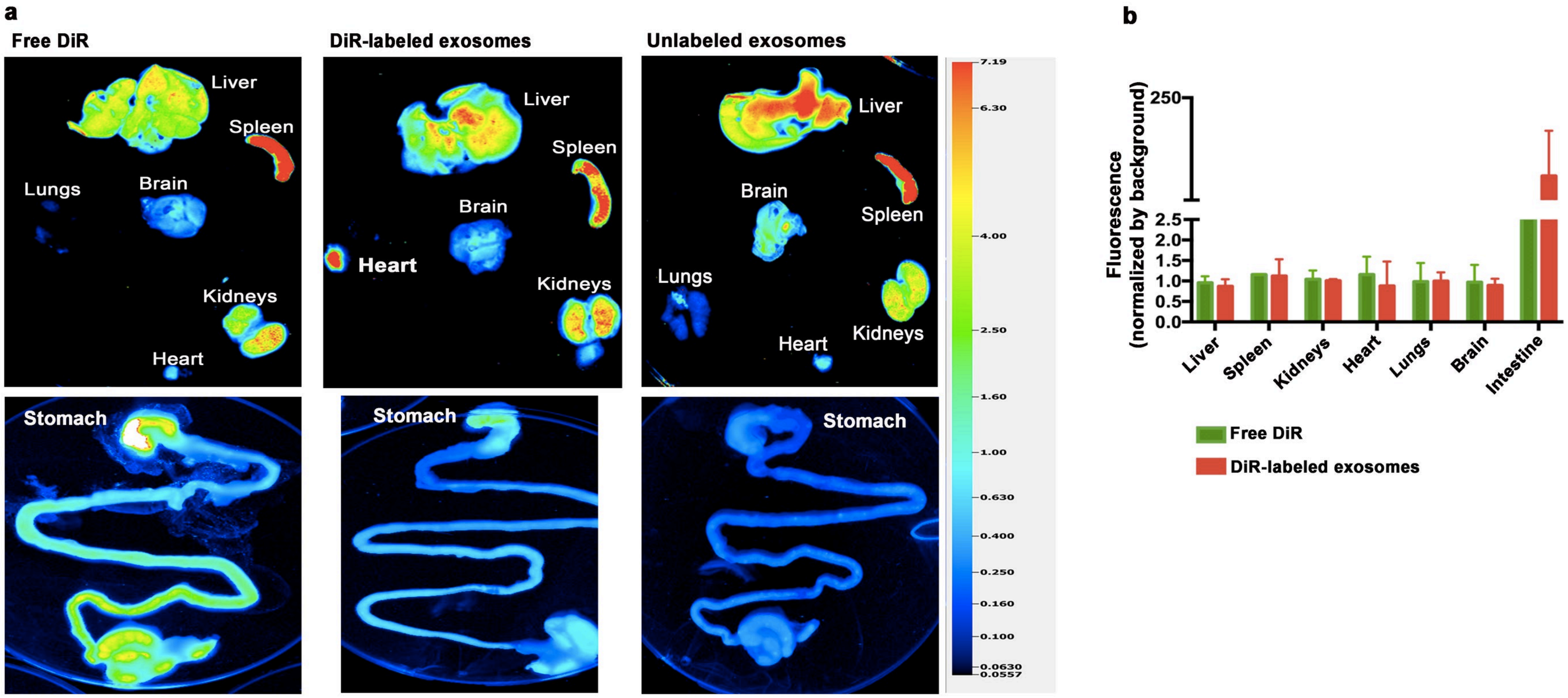

Supplementary Fig. 2 Distribution of bovine milk exosomes in male Balb/c mice. **a** Fluorescence signal in excised organs 24 hours after oral gavage of free DiR, unlabeled and DiR-labeled exosomes (1 *x* 10^{12}/g body weight). **b** Densitometry analysis of excised tissues of DiR-labeled exosomes 24 hours after oral gavage, normalized for plate background ($p > 0.10$, n = 4)**.** Panels in this figure were assembled from multiple independent images; individual images in the grouped figure are separated by white space.

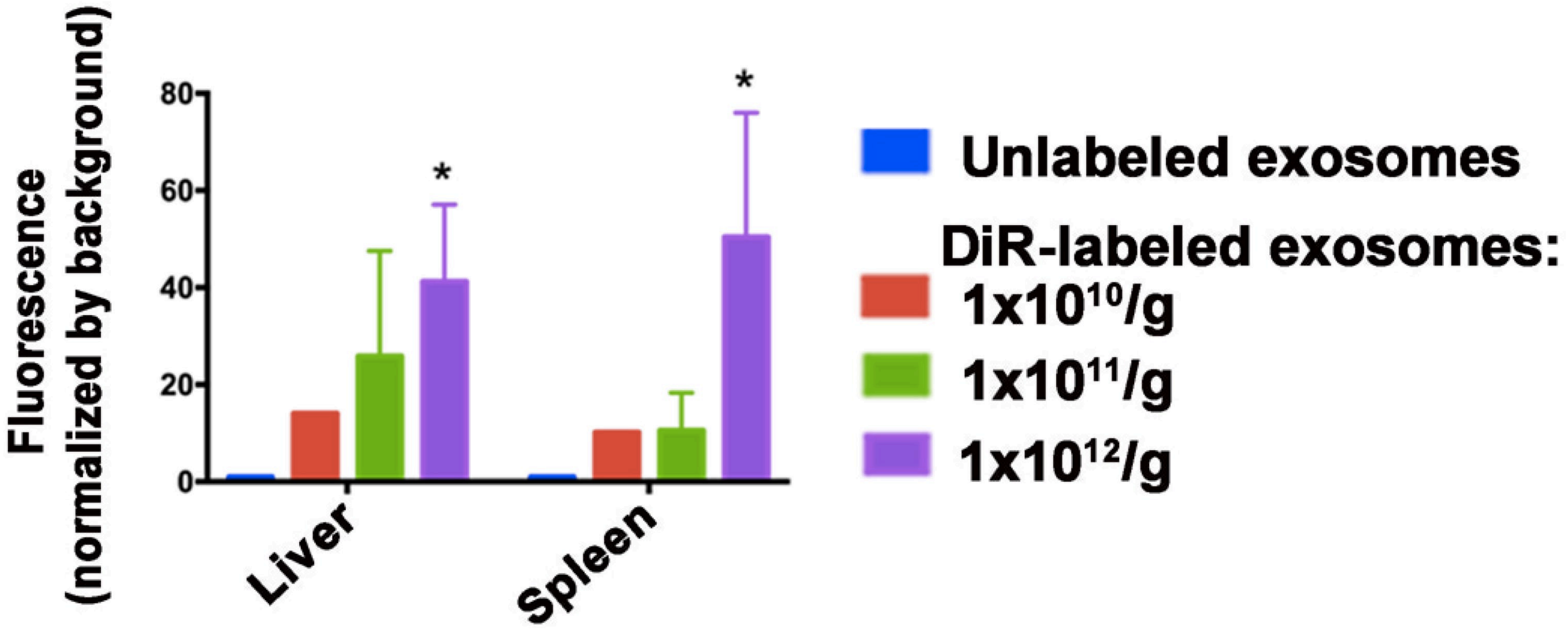

Supplementary Fig. 3 Mean of densitometry analysis shown in Fig. 1g. Mean of excised tissues after intravenous injection of unlabeled or DiR-labeled exosomes ($1\ x\ 10^{10}$/g, $1\ x\ 10^{11}$/g, $1\ x\ 10^{12}$/g body weight) shown in Fig. 1g (*$p < 0.05$ by one sample *t*-test, n = 4).

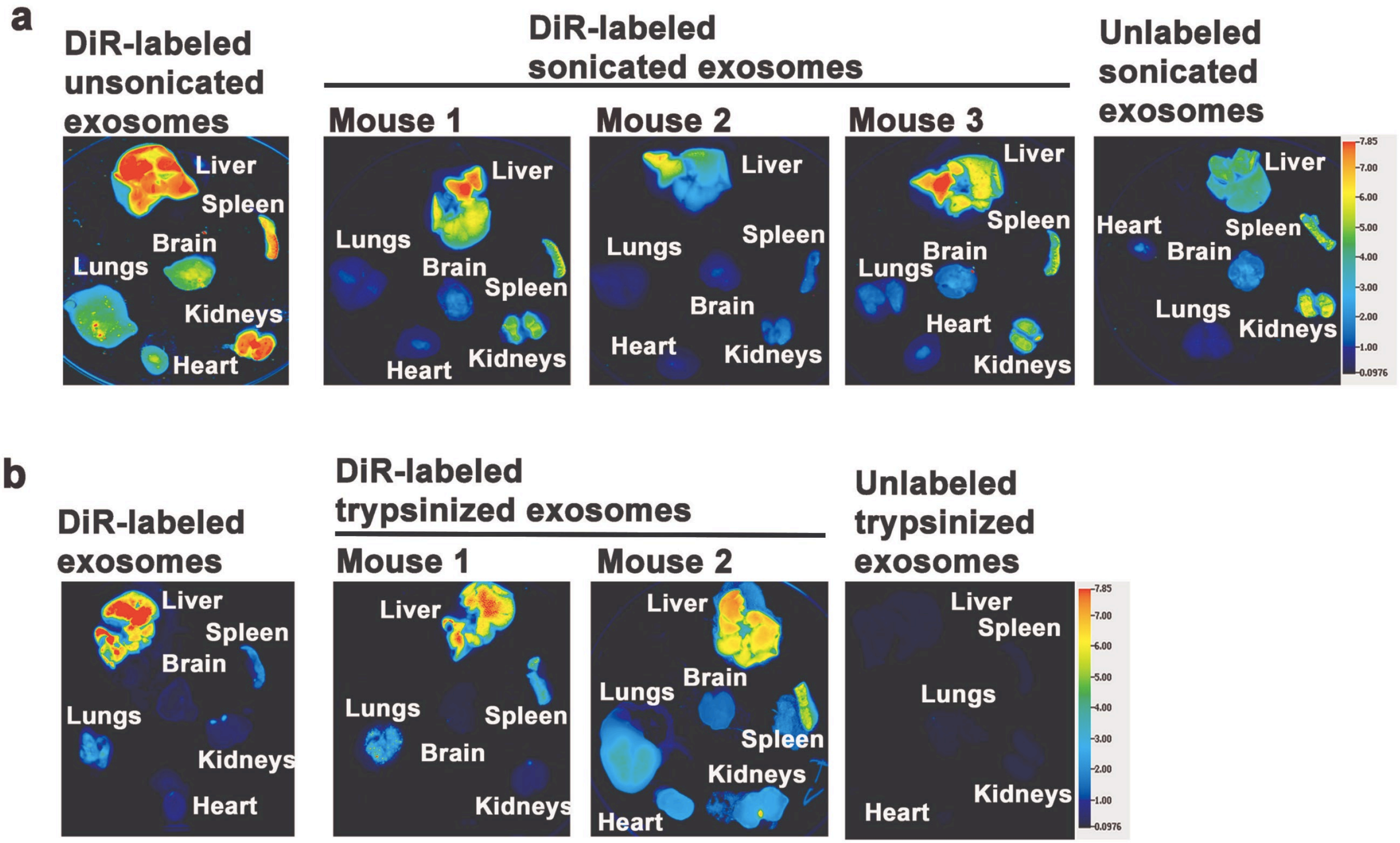

Supplementary Fig. 4 Distribution of ultrasonicated and trypsinized exosomes in Balb/c mice. **a** Fluorescence signal in tissues excised from Balb/c mice 24 hours after administration of unsonicated exosomes (DiR-labeled or unlabeled) and ultrasonicated (DiR-labeled or unlabeled) by oral gavage (1 x 10^{12}/g body weight, n = 3). **b** Distribution of trypsinized DiR-labeled exosomes (1 x 10^{12}/g body weight) in tissues excised 24 hours after oral gavage (n = 2). Panels in this figure were assembled from multiple independent images; individual images in the grouped figure are separated by white space.

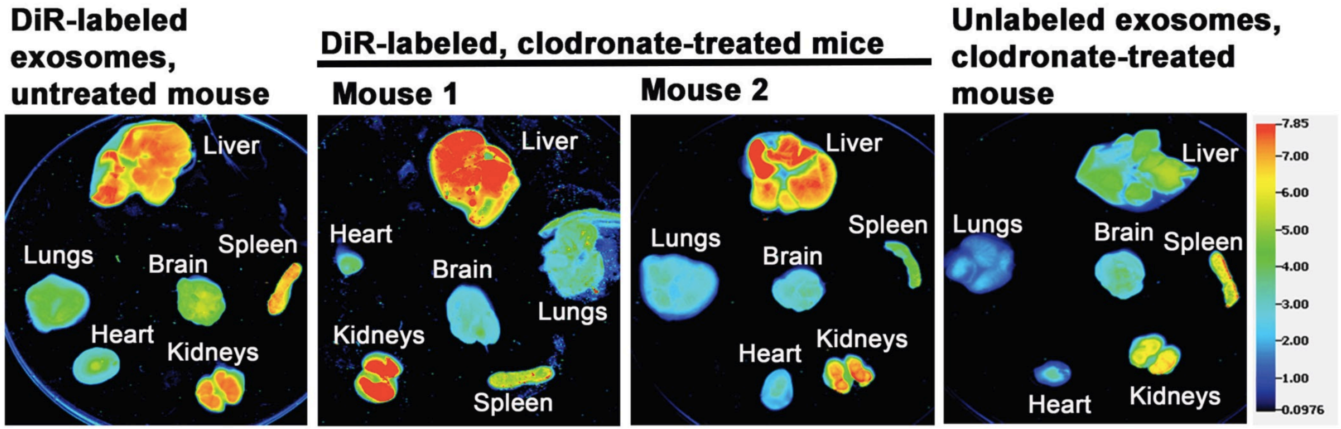

Supplementary Fig. 5 Distribution of exosomes in macrophage-depleted mice. Balb/c mice were treated with clodronate (150 ul) by intraperitoneal injection to ablate endogenous macrophage populations. Unlabeled or DiR-labeled exosomes were administered by oral gavage 24 hours after clodronate treatment, and tissues were harvested 24 hours after exosome administration for fluorescence analysis. Panels in this figure were assembled from multiple independent images; individual images in the grouped figure are separated by white space.

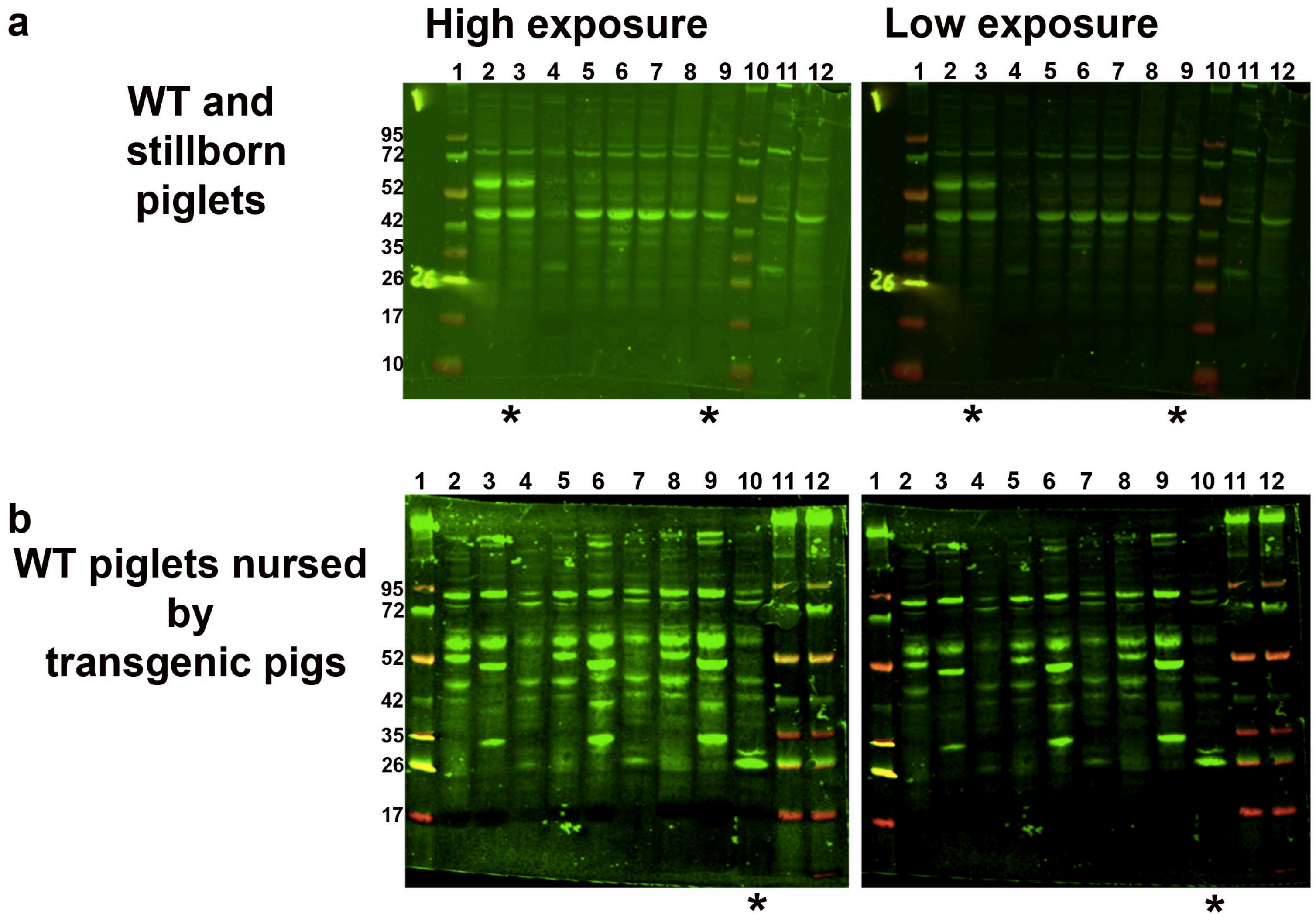

Supplementary Fig. 6 Presentation of the full gels of the lanes in Fig. 2b at high and low exposure. Lanes labeled with an * are those shown in Fig. 2b. **a** Western blot analysis of ZsGreen1 in protein extracts from WT piglets nursed by a WT sow for 17 days and from stillborn WT piglets from a transgenic sow. Lanes: 1, marker; 2, WT piglet 1, brain back right; 3, WT piglet 1, cerebellum (shown as lane 2 in Fig. 2b); 4, stillborn piglet 1, liver; 5, stillborn piglet 1, brain back right; 6, stillborn piglet 1, brain back left; 7, stillborn piglet 1, brain front right; 8, stillborn piglet 1, brain front left; 9, stillborn piglet 1, cerebellum (shown as lane 3 in Fig. 2b); 10, marker; 11, stillborn piglet 2, liver; 12, stillborn piglet 2, cerebellum. **b** Western blot analysis of ZsGreen1 in protein extracts from WT piglets 1, 2 and 3 nursed by a transgenic ZsGreen1 sow for 17 days. Lanes: 1, marker; 2, piglet 1, spleen; 3, piglet 1 liver; 4, piglet 1, cerebellum; 5, piglet 2, spleen; 6, piglet 2, liver; 7, piglet 2, cerebellum; 8, piglet 3, spleen; 9, piglet 3, liver; 10, piglet 3, cerebellum (shown as lane 4 in Fig. 2b); 11, marker; 12, marker.

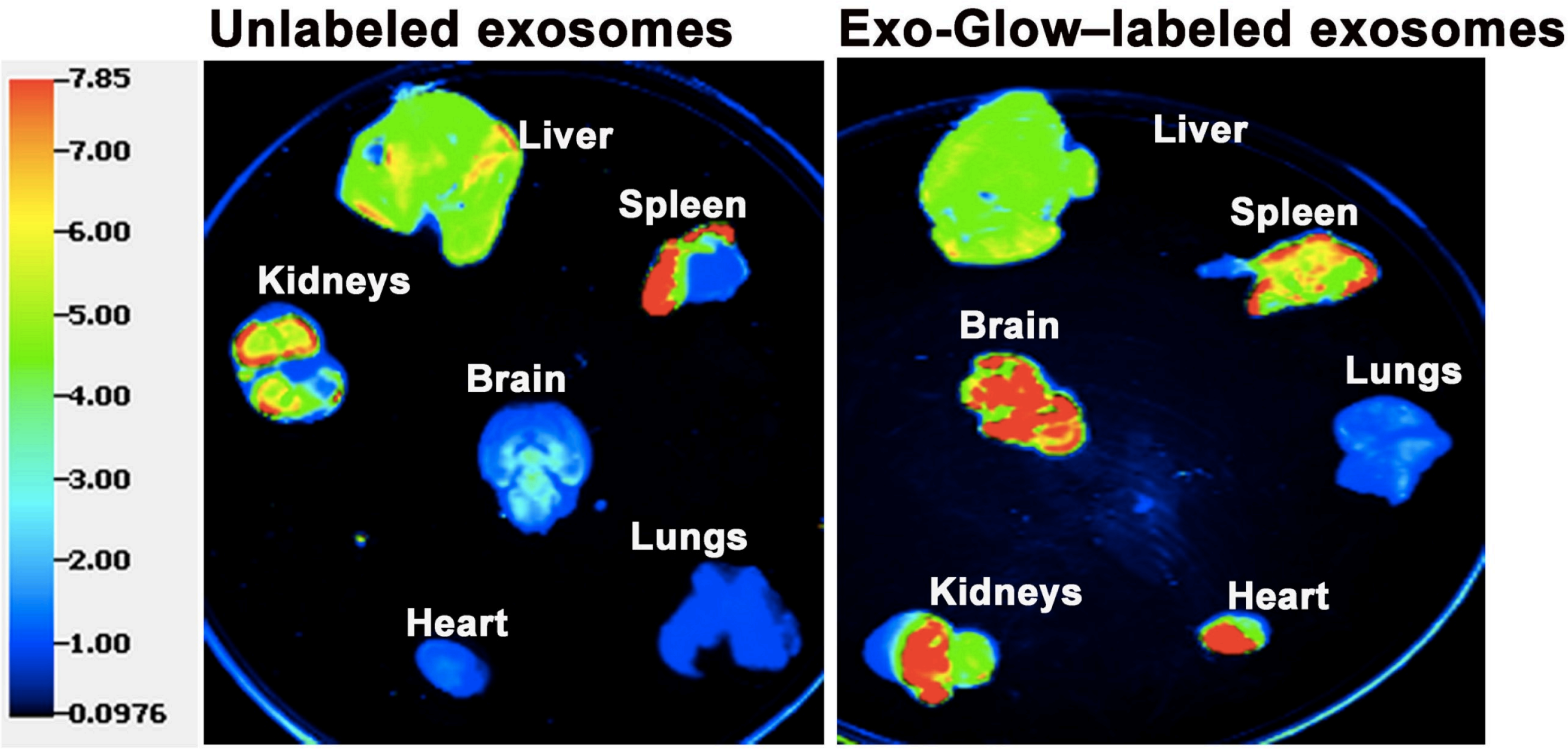

Supplementary Fig. 7 Distribution of single stranded RNA following intravenous injection of bovine milk exosomes containing Exo-Glow–labeled RNA in Balb/c mice. Distribution of unlabeled and Exo-Glow–labeled RNA 2 hours after intravenous injection of unlabeled or Exo-Glow-labeled bovine milk exosomes (1 *x* 10^{12}/g body weight). Panels in this figure were assembled from multiple independent images; individual images in the grouped figure are separated by white space.

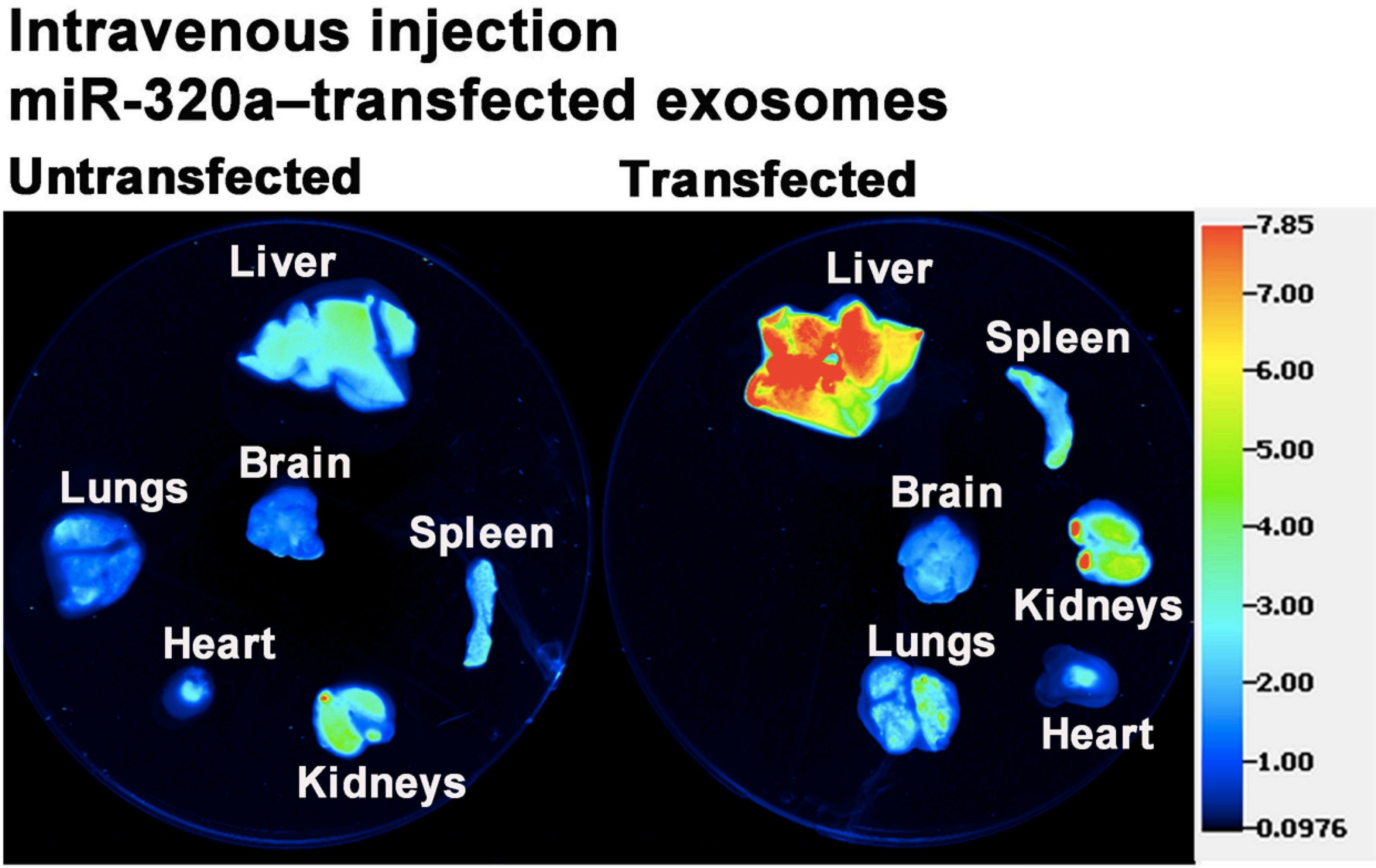

Supplementary Fig. 8 Distribution of fluorophore-labeled miRNA-320a transfected into bovine milk exosomes and administered to Balb/c mice. Fluorescence signal of excised tissues from female mice 3 hours after intravenous injection with synthetic IRDye-labeled miR-320a, transfected into milk exosomes (1 x 10^{12}/g body weight).

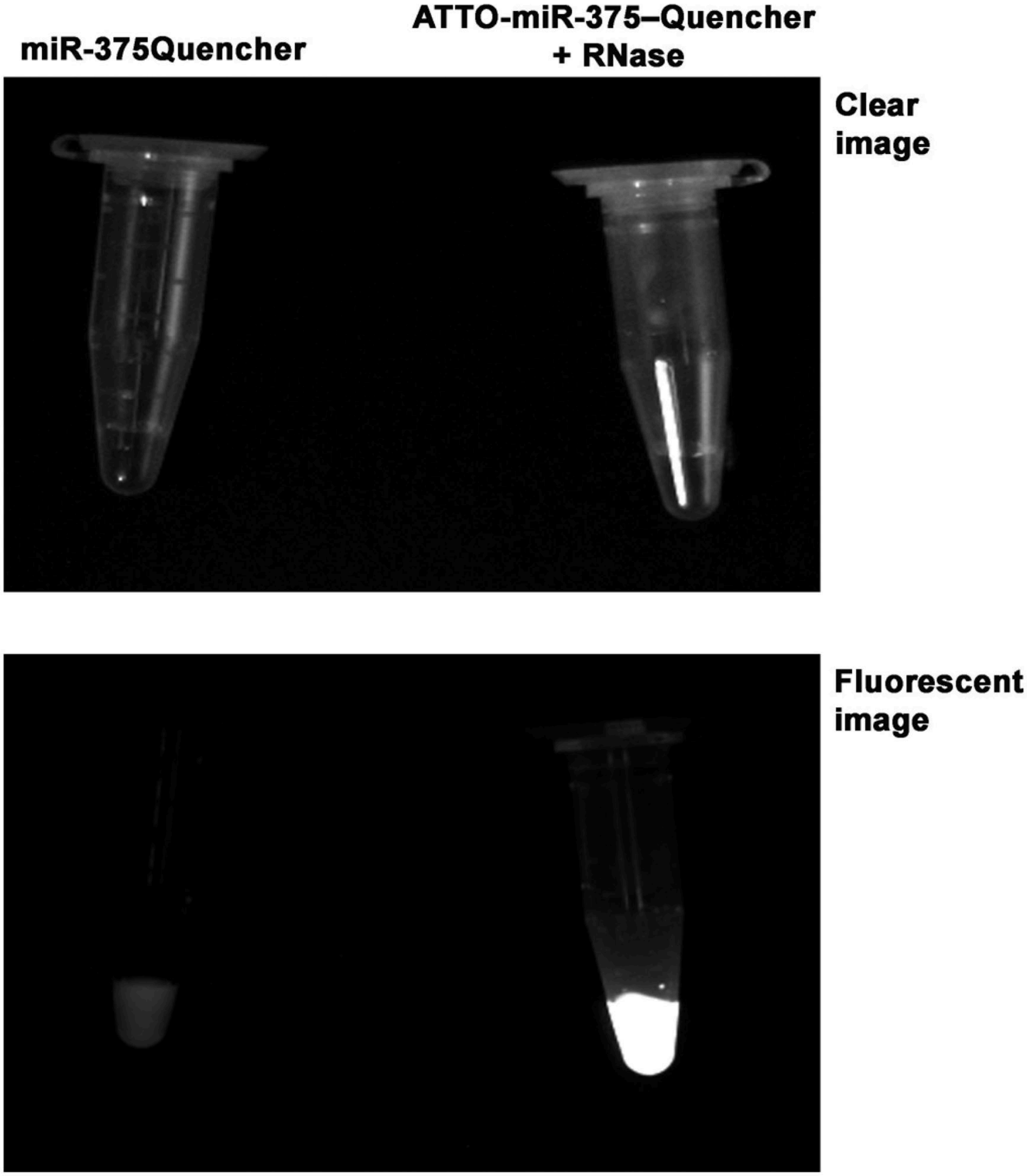

Supplementary Fig. 9 RNase treatment of miR-375 covalently labeled with both fluorophore and quencher. Fluorescence analysis of with untreated labeled-miR-375 and synthetic miR-375 labeled with fluorophore (5ATTO633N) and quencher (3IAbRQSp) prior to and after treatment with RNase. Panels in this figure were assembled from independent images; individual images in the grouped figure are separated by white space.

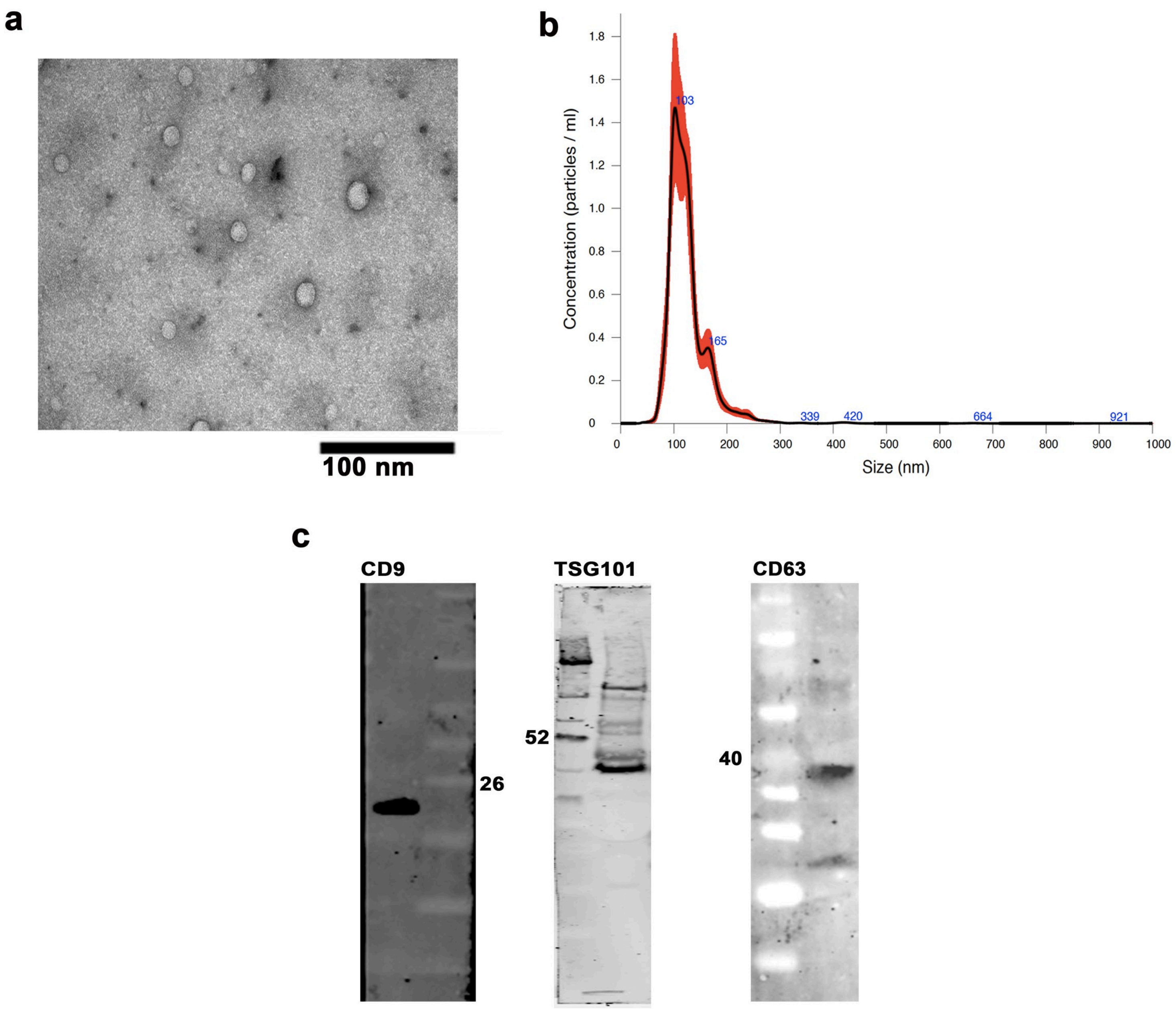

Supplementary Fig. 10 Exosome authentication. Exosomes were authenticated by transmission electron microscopy (**a**), NanoSight NS300 nanoparticle tracking analysis (**b**), and western blot analysis (**c**).
